# Prenatal Exposure to Favorable Social and Environmental Neighborhood Conditions Is Associated with Healthy Pregnancy and Infant Outcomes

**DOI:** 10.3390/ijerph18116161

**Published:** 2021-06-07

**Authors:** Allison A. Appleton, Betty Lin, Elizabeth A. Holdsworth, Beth J. Feingold, Lawrence M. Schell

**Affiliations:** 1 Department of Epidemiology and Biostatistics, University at Albany School of Public Health, Rensselaer, NY 12144, USA; lmschell@albany.edu; 2 Department of Psychology, University at Albany College of Arts and Sciences, 1400 Washington Street, Albany, NY 12222, USA; blin6@albany.edu; 3 Department of Anthropology, University at Albany College of Arts and Sciences, 1400 Washington Street, Albany, NY 12222, USA; eholdsworth@albany.edu; 4 Department of Environmental Health Sciences, University at Albany School of Public Health, Rensselaer, NY 12144, USA; bfeingold@albany.edu

**Keywords:** developmental origins of health and disease, child opportunity index, pregnancy health, birth outcomes, social and environmental determinants of health

## Abstract

Neighborhood and individual level risks commonly co-occur for pregnant women and may cumulatively contribute to birth outcomes. Moreover, the relationship between favorable social and environmental neighborhood conditions and perinatal outcomes has been understudied. This study considered the accumulated impact of prenatal exposure to positive neighborhood social, environmental, and educational conditions in relation to maternal health during pregnancy and birth size outcomes. In a prospective study of a multi-ethnic and socioeconomically diverse cohort (*n* = 239) of pregnant women and their infants, neighborhoods were characterized by the Child Opportunity Index (COI), a census-tract composite indicator representing favorable social, environmental, and educational community conditions. Adjusted generalized estimating equations showed that favorable neighborhood conditions promoted the growth of longer and heavier infant bodies, and reduced the risk of intrauterine growth restriction. The associations were stronger for female versus male infants, though not significantly different. Moreover, COI was associated with better maternal mental health and diet during pregnancy; diet significantly mediated the association between COI and birth size outcomes. This study underscores the importance of considering the accumulated benefit of neighborhood assets for maternal and infant health. Interventions that capitalizes on the full range of contextual assets in which mothers live may promote pregnancy health and fetal growth.

## 1. Introduction

Gestation is a critical period of development, when risk and protective exposures can influence health across the life course [1,2]. Birth size outcomes, including birth weight, length, head circumference, and intrauterine growth restriction, have been commonly examined as indicators of the quality of the intrauterine environment [1], and may also be sentinels for future health risk [3,4]. Moreover, patterning in prenatal exposure and birth size associations are often sex-specific [5], with male and female developing body systems differentially affected by social and environmental exposures [6]. The determinants of birth size outcomes are varied, and include neighborhood conditions [7] as well as individual level maternal risk factors [8]. Given the range of gestational exposures that can contribute to birth size outcomes, research is needed that considers the interrelationships and accumulated impacts of multiple exposures working simultaneously at different scales.

Neighborhood conditions, including social and economic disadvantage [9], aspects of the built environment [10], and presence/proximity to greenspace and toxicants [11,12], are associated with birth size, with adverse conditions generally shown to contribute to smaller infant size and earlier gestational age at birth. The pathways linking such neighborhood conditions and birth outcomes are not fully understood. Maternal health during pregnancy may be one way in which neighborhood conditions become biologically embedded to affect infant outcomes. Several studies have shown that prenatal depression [13], diet [14,15], smoking [16], body mass and weight gain [17,18,19], and pregnancy morbidities [8] can influence birth outcomes, with some evidence that these factors may mediate associations between neighborhood conditions and birth outcomes [20,21,22,23]. However, research evaluating links between neighborhood conditions, maternal health, and birth outcomes has tended to focus on singular aspects of the neighborhood context (e.g., either socioeconomic or environmental conditions) or a single type of mediator (e.g., pregnancy conditions versus depression). Thus, the understanding about the full spectrum of potential pathways linking neighborhood exposures to birth outcomes is incomplete. Such pathways need to be evaluated in order to offer intervention points that can curb the ill effects of neighborhood risks and promote healthy birth outcomes.

Social and environmental risks commonly co-occur for pregnant women and children, particularly among vulnerable populations [24,25,26]. As such, focusing on the independent effects of one type of exposure may lead to underestimated or mis-specified associations [27]. Researchers adopting an interdisciplinary perspective often find that the combined effect of social and environmental risks are stronger than when considered individually [24,26], including for birth outcomes [28,29]. However, as this is an emergent area of research, further characterization of the combined influence of neighborhood social and environmental factors for birth outcomes is needed. The Child Opportunity Index (COI) is a multidimensional indicator of neighborhood context that includes information on resources and favorable community conditions that can promote health and enable children to thrive [30]. Positive factors, such as the COI, do not simply reflect the absence of risk, but instead are independent attributes or assets that can enhance health and promote resilience [31]. As a composite metric, the COI includes positive factors from social, environmental, and educational neighborhood domains. Recent work finds that a higher neighborhood COI exerts a protective effect in relation to individual level child physical health and cortisol levels [32], pediatric acute care utilization [33], and youth cardiometabolic health [34,35]. The COI has not been analyzed in association with pregnancy health and birth size. A developmental origins of health and disease (DOHaD) perspective would suggest that better social and environmental neighborhood quality during pregnancy may enhance maternal health, which in turn may support a healthy intrauterine environment and optimal fetal growth. However, as most DOHaD work considers individual level prenatal risks rather than assets, the possibility has not been evaluated.

This study examined the accumulated impact of positive neighborhood social and environmental factors, as measured by the COI, in relation to maternal health during pregnancy and birth size outcomes. We hypothesized that higher COI scores in the prenatal period would be associated with healthier birth size outcomes. Also, as much DOHaD work notes sex-specific associations for prenatal exposures and birth outcomes [5,6], we hypothesized that associations would be differentially patterned for males and females. We also hypothesized that maternal health during pregnancy would mediate the association between COI and birth size. We considered a range of potential mediators that could plausibly link neighborhood context with birth outcomes, including maternal weight gain and body size metrics, pregnancy complications, smoking, diet, and depression. We tested our hypotheses using data from a prospective birth cohort study and controlled for a robust set of individual level covariates. Moreover, where most neighborhood research examines the contextual effects in major metropolitan areas, we focused on a unique geography that is comprised of a set of small cities, suburban regions, and rural areas in and around Albany, NY, which are collectively known as the Capital District in upstate New York. This study is the first to consider the combined influence of positive social and environmental neighborhood attributes during pregnancy in relation to maternal health and birth outcomes.

## 2. Materials and Methods

### 2.1. Study Population

Participants were part of the Albany Infant and Mother Study (AIMS), a prospective observational cohort study of pregnant women and their infants born at Albany Medical Center (Albany, NY, USA) [36]. English speaking women, 18–40 years old, with singleton pregnancies were eligible to participate. Women enrolled on average at 27 weeks gestation at an outpatient obstetrics clinic. At the prenatal enrollment visit, participants completed questionnaires that assessed demographic factors, health histories, pregnancy behaviors, psychosocial, and environmental factors. Maternal residential address at the pregnancy enrollment visit was also collected. Following the birth, study physicians conducted a structured medical record review to obtain clinical information on maternal health, delivery, and infant characteristics, including birth outcome information and infant sex. Three-hundred mother–infant pairs enrolled, with *n* = 290 eligible participants completing the prenatal and birth assessments. The present analysis includes the *n* = 239 with complete information on residential address during pregnancy, maternal health, birth outcomes, and covariate information. Protocols and informed consent documents were approved by institutional review boards at Albany Medical Center and the University at Albany State University of New York.

### 2.2. Measures

Neighborhood Child Opportunity Index. Participant residential addresses during pregnancy were geocoded in ArcMap version 10 (ESRI, Redlands, CA, USA) using the building geocoding function and the US Address—Single House address locator. These geocoded addresses were linked to 2015 census tracts using QGIS. Neighborhood conditions for each participant were measured using the Child Opportunity Index 2.0 [30,37]. This index uses a compilation of multi-sourced nationally representative data to characterize the following three domains of neighborhoods by census tract: education, health and environment, and social and economic opportunities. Each domain includes several component parts that capture distinct features of the neighborhood (Supplemental Table S1). Educational opportunity includes eleven indicators concerning early childhood education, elementary education, secondary and postsecondary education, and educational and social resources. The health and environment domain includes ten indicators concerning healthy physical environments, greenspace, toxic exposures, and health resources. The social and economic domain includes eight indicators concerning economic opportunities, as well as economic and social resources in a community. Complete information on COI component parts, derivation and validation are available at www.diversitydatakids.org (accessed on 10 November 2020).

Continuous COI scores for each census tract were used in this analysis, with higher scores representing more neighborhood opportunity. This COI score reflects a rank order of all 72,000 neighborhoods in the United States according to child opportunity, weighted by the number of children in each census tract, resulting in 100 rank-ordered groups. The COI scores range from 1 to 100 (1 = 1% of children living in lowest opportunity neighborhoods; 100 = 1% of children living in highest opportunity neighborhoods). We also examined the distribution of neighborhood-level child opportunity according to quintiles (very low, low, moderate, high, and very high opportunity), which were derived according to national distributions of COI scores. All COI variables used in the analysis were from 2015, the year in which the majority of AIMS participants were pregnant and/or the immediate time preceding the pregnancy.

Maternal characteristics. Demographics included self-reported maternal age and race/ethnicity (white versus black/Hispanic/other). Maternal pregnancy health factors included weight and body size metrics, pregnancy complications, behaviors, and depression. Specifically, the weight and body size metrics included pre-pregnancy body mass index (weight in kilograms/height in m^2^; calculated from self-reported pre-pregnancy height and weight provided at the enrollment visit) and weight gain in pregnancy (abstracted from medical records and dichotomized according to whether or not too much weight was gained given pre-pregnancy BMI following American College of Obstetricians and Gynecologists standards [17]. Pregnancy complications were abstracted from medical records and summarized into an index that included pregnancy conditions (gestational diabetes, preeclampsia, eclampsia), placental abnormalities (abruption, previa, accreta, marginal bleed), gynecologic bacterial infections (group B streptococcus, chorioamnionitis), and preterm premature rupture of the membranes, with higher scores indicating more pregnancy complications.

Maternal behaviors included self-reported smoking during pregnancy (yes/no), and diet during pregnancy. Diet was assessed in pregnancy with a 25-item food frequency questionnaire [38,39]; a western diet sum score was derived, with higher scores indicating more frequent consumption of foods from western categories (e.g., red meats, processed meats, refined grains, high-fat dairy products, potatoes, sugar sweetened beverages). Finally, depressive symptoms during pregnancy were measured with the Edinburgh Postnatal Depression Scale (EPDS; α = 0.87), a self-report scale that has been validated for use among pregnant and postpartum populations and focuses on the cognitive and affective features of depression rather than somatic complaints [40]. The EPDS assesses the intensity of depressive symptoms in the past week, with higher scores indicating more depressive symptoms.

Infant characteristics. Anthropometric measurements of the newborn were conducted in the delivery room by trained clinical staff using standard protocols and instrumentation. Information on gestational age at birth (weeks), birth weight (kilograms), birth length (centimeters), head circumference (centimeters) were abstracted from medical records. In addition, a cephalization index ((head circumference cm/birth weight g)*100)) was derived. This index reflects a certain type of intrauterine growth restriction that is characterized by asymmetry (e.g., larger heads proportional to body size). This asymmetry can signal potential brain sparing during gestation and offspring neurodevelopmental vulnerability [41,42]. Higher cephalization index scores reflect greater asymmetry in fetal growth. Size for gestational age was calculated according to Fenton standards [43], with small (SGA) and large for gestational age (LGA) reflecting infants born at the bottom 10th percentile and top 90th percentile for weight for a given gestational age, respectively. Infant sex was abstracted from medical records (male/female).

### 2.3. Analytic Plan

First, those excluded from the complete case analysis were compared to those included in the analytic sample according to maternal sociodemographic factors and COI scores via independent t-tests and Chi-square tests. Descriptive statistics were generated for all study variables, and bivariate associations were assessed via Pearson’s correlations. Associations between neighborhood COI and infant outcomes were assessed with generalized estimating equations (GEE) with exchangeable correlation structures, adjusted for covariates. Because observations were not independent due to clustering at the census tract level, we used GEE, which provided parameter estimates, *p*-values, and confidence intervals that accounted for this correlated data structure. This GEE modeling approach is consistent with other recent work studying the association between COI and child outcomes [33]. To test for sex patterning in COI and infant outcome associations, we followed standard epidemiologic approaches for testing for sex-specific effects by adding an interaction term to the final models, and stratifying COI and birth outcome models by infant sex [44,45]. To obtain estimates of effect size, Cohen’s d statistics were calculated for birth outcomes for those living in neighborhoods characterized by “very low” versus “very high” child opportunity. Associations between neighborhood COI and maternal health during pregnancy were also assessed with GEE, adjusted for covariates. For maternal health and infant outcomes that were found to be significantly associated with COI, additional analyses were conducted to further contextualize the associations. First, Sobel tests [46,47] were conducted to determine whether maternal pregnancy health variables mediated the association between COI and infant outcomes. Finally, in order to describe whether a particular COI domain (educational, health and environmental, or social an economic opportunity) was driving the associations between the overall COI score with study outcomes, we fit separate models for each domain with study outcomes via GEE. Models testing the associations between COI and infant outcomes were adjusted for factors that can influence birth size including maternal age, race/ethnicity, smoking during pregnancy, child sex, and gestational age at birth. Models testing the associations between COI and maternal health were adjusted for maternal age and race/ethnicity. Maternal education was not included as a covariate in any model to avoid collinearity; several component parts of the COI include indicators of education and education attainment. Statistical significance was determined by *p*-values < 0.05 and 95% confidence intervals.

## 3. Results

There were no significant differences between those included and excluded from the complete case analysis in terms of maternal age, race, education attainment (high school or less vs. higher) and child sex. Those excluded were more likely to have lower COI scores (t = 2.54, *p* = 0.01). The characteristics of the study population included in this analysis are listed in Table 1. Pregnant participants were on average 28.6 (SD = 5.57) years old at enrollment, and approximately half were white. The average pre-pregnancy BMI was 28.87 (SD = 8.67), and half of the participants had a high degree of weight gain in pregnancy. The average maternal depressive symptom score was 8.67 (SD = 5.44), with 22.2% meeting the threshold for clinical depression during pregnancy. The participants experienced on average less than one pregnancy complication (range 0–4 complications). Approximately 14% smoked during pregnancy, and the average western diet sum score was 40 (SD = 15.29; or approximately 17 servings of western style foods per week). Approximately an equal number of the infants were male and female. The infants were born on average at 39 weeks gestational age (SD = 1.91; range = 28.86–42.0 weeks), weighed 3.26 kg (SD = 0.62), were 48.77 cm long (SD = 3.18), and had an average head circumference of 33.47 cm (SD = 2.17) at birth. There was a low prevalence of small and large for gestational age infants in the sample, and the average cephalization index scores suggest normative fetal growth. The average COI score was 45.69 (SD = 32.80), indicating that the participants in this region lived in neighborhoods characterized by lower child opportunity compared to neighborhoods nationwide.

**Figure 1:**
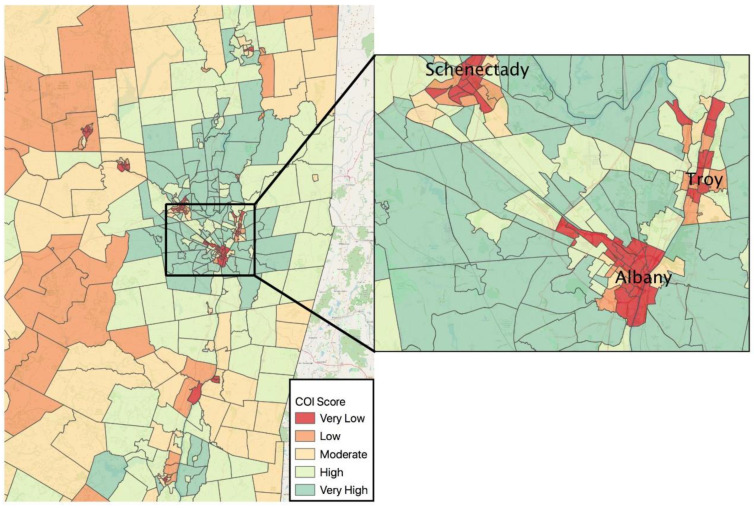
Maps of Child Opportunity Index (COI) scores by Census tract for the New York State Capital District. The left map includes the urban, suburban, and rural areas participants inhabited during pregnancy, as well as neighboring census tracts. All regional tracts were included so as to protect confidentiality of participants as some tracts included less than five people. Very low COI scores were heavily concentrated in urban areas, with low and moderate COI in surrounding rural regions, and very high COI concentrated in the suburbs of urban centers. The right map zooms in on the urban center of the Capital District, which encompasses the cities of Albany, Schenectady, and Troy, which is where the majority of participants lived.

The distribution of the COI scores in the study catchment area and across New York’s Capital District are shown in Figure 1. This region is a unique amalgamation of three small cities, suburban bedroom communities, and rural regions that together comprise the Capital District. Approximately half (49%) of the participants lived in neighborhoods characterized as “very low” and “low” opportunity during pregnancy; 22% lived in neighborhoods with “very high” opportunity. “Low” and “very low” opportunity neighborhoods were concentrated in the urban centers of the cities of Albany, Schenectady, and Troy; 57% of the AIMS participants lived in these cities. Higher opportunity was concentrated in suburban regions. Rural communities tended to have a mix of “low” or “moderate” opportunity.

**Figure 2:**
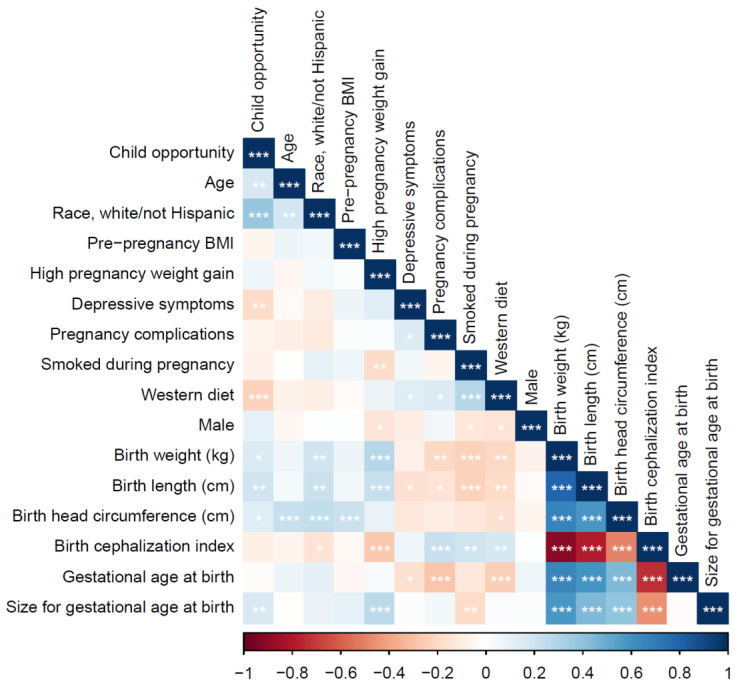
Correlations among study variables.

The bivariate associations between the study variables are listed in Figure 2. Overall, higher COI scores were significantly correlated with older maternal age, white/non-Hispanic race, fewer depressive symptoms during pregnancy, less adherence to a western diet during pregnancy, as well as higher birth weight, longer birth length, greater head circumference, and appropriate size for gestational age at birth. All maternal pregnancy health factors were significantly correlated with the infant outcomes; patterning was in the expected directions, with a greater maternal pregnancy risk associated associations between neighborhood level child opportunied with riskier infant outcomes.

The adjusted associations between the COI scores and infant outcomes are listed in Table 2. Higher COI scores were significantly associated with higher birth weights and longer birth lengths, and marginally associated with lower infant cephalization scores (which indicates less asymmetry in fetal growth/normative intrauterine growth). The head circumference and size for gestational age were not associated with the COI. The results were substantively comparable for all of the outcomes without controlling for gestational age (see Supplemental Table S2). The sex-stratified models showed some patterning in the COI and infant outcome associations. Specifically, while the association between COI and birth weight was similar for males and females, the associations between COI and birth length and cephalization were significant for females, but not for males. Also, the risk of being born small for gestational age was lower according to higher COI scores for females, but not for males. However, none of the interaction terms testing the sex-specific associations were statistically significant (results not shown). Cohen’s d statistics indicated that the effects of COI on birth outcomes for those living in “very high” versus “very low” child opportunity neighborhoods were small to moderate in size (*d*_birthweight_ = 0.36, *d*_birthlength_ = 0.53, *d*_headcircumference_ = 0.42, *d*_cephalization_ = 0.20).

The adjusted associations between the COI scores and maternal health during pregnancy are listed in Table 3. Higher COI scores were significantly associated with fewer depressive symptoms and lower western diet scores, adjusting for maternal age and race/ethnicity. Pregnancy complications, pre-pregnancy BMI, high weight gain, and smoking during pregnancy were not associated with the COI scores in the adjusted models.

Given the significant associations with COI, additional analyses were conducted to determine whether maternal depressive symptoms or western diet mediated the associations with birth weight, birth length, and cephalization. Sobel tests determined that the mediated effect of a western diet in the associations between COI and birth weight (t = 2.33, SE = 0.33, *p* = 0.02) and birth length (t = 1.99, SE = 0.002, *p* = 0.045) were significantly different from zero. No evidence of mediation was observed for western diet and cephalization index (*p* = 0.14), or for depressive symptoms and any outcome (all *p* > 0.17). See Supplemental Table S3 for Sobel test inputs and results.

The adjusted associations between each domain of the COI (educational, health, environmental, social and economic) and maternal and infant outcomes are shown in Table 4. Each domain of the COI was associated with birthweight and birth length; all of the domains, but social and economic, were associated with cephalization. Each domain of the COI was associated with maternal western diet scores; all of the domains, but educational, were associated with maternal depressive symptoms.

## 4. Discussion

This is the first study to consider the accumulated impacts of positive neighborhood educational, social, and environmental attributes in relation to pregnancy health and birth outcomes. The results indicated that neighborhood-level opportunity was cumulatively associated with healthier infant birth size and maternal health during pregnancy, even after adjusting for individual level maternal age and race and ethnicity, as well as other known contributors to birth size outcomes including smoking during pregnancy, child sex, and gestational age at birth. The results also suggested that maternal diet during pregnancy mediated the associations between neighborhood opportunity and birth size. These findings were particularly noteworthy as we considered the role of neighborhood opportunity in maternal and child health outcomes in a unique geography that includes a heterogeneous mix of small cities, suburban neighborhoods, and rural areas. Disparities and assets in regions like this tend to be under-characterized in the literature.

The associations between COI and infant birth size outcomes indicated that positive aspects of the neighborhood social and physical environment promoted the growth of longer and heavier infant bodies, but not larger head circumferences, which suggests normative symmetry in fetal growth, as well as the absence of restricted growth and brain sparing during fetal development. The associations were adjusted for gestational age, and thus do not reflect a spatial distribution of prematurity and term births in the region. These findings are novel and highlight that just as adverse gestational exposures can contribute to poor birth outcomes, positive factors can likewise influence the developing fetus and promote healthy growth. The results from the sex-stratified models suggested that the positive effect of COI on birth size outcomes may be stronger for female infants than for males. However, the interaction tests for infant sex and COI did not reach statistical significance. While much DOHaD work observes sex-specific patterning in prenatal exposure and infant outcome associations, we cannot conclusively determine if the effect of COI was different for males and females in this sample. More work with larger sample sizes is needed to test for the sex-specific effects of prenatal COI exposure on birth outcomes.

To our knowledge, only one study has considered the effect of combined (adverse) social and environmental exposures during pregnancy at the neighborhood level in relation to birth size outcomes [48]. In that study of 879 U.S. mother–infant pairs, composite indicators of neighborhood environmental hazards (e.g., census tract level air pollution, traffic density, waste sites), social determinants of health (e.g., census tract level crime rates, poverty, hospitalization rates), and an index combining both sets of neighborhood exposures during pregnancy were considered in relation to birth weight and neonate body composition. Controlling for individual level characteristics, the combined neighborhood hazard index showed the greatest reduction in birth weight and neonatal fat mass than the environmental and social hazard domains did independently. Our findings are congruent with this study, and taken together, this emergent area of research shows that social and environmental risks and assets can jointly contribute to birth outcomes.

Greater neighborhood opportunity was associated with fewer maternal depressive symptoms and less adherence to a western diet during pregnancy. The Sobel tests indicated that maternal diet significantly mediated the association between the COI and birth weight and birth length in this sample. These findings suggest that favorable social and environmental neighborhood conditions may help promote maternal mental health and healthy dietary practices during pregnancy, which, at least for diet, has implications for birth size. One component of the COI score was access to healthy foods (a subpart of the health and environment domain). This neighborhood characteristic was negatively correlated with a western diet (r = −0.11, *p* = 0.05), suggesting that having greater access to healthy foods directly contributed to less adherence to a western diet during pregnancy. We also found that diet during pregnancy was associated with each COI domain, suggesting that educational, social and economic features of the neighborhood (apart from food access) can also promote healthy eating during pregnancy. For depression, we did not observe a mediation effect between COI and birth size outcomes, despite finding that COI contributed to better maternal mental health. It may be that in the prenatal period, the effects of COI may be relayed to the fetus through dietary intake, which can directly affect physical growth in utero, whereas the mental health pathway linking COI to child outcomes may come into play postnatally when social and emotional interactions with caregivers is paramount. We have initiated a follow-up protocol to characterize the AIMS cohort in early childhood; our future work will be able to study this question specifically.

Given the associations with COI and diet, the lack of associations between COI and pre-pregnancy BMI and weight gain were somewhat surprising. The null associations found here could be due in part to ceiling effects; half of the sample had high pregnancy weight gain and the average pre-pregnancy BMI reflected overweight and near-obese thresholds. There may not have been sufficient variability in the maternal weight metrics to detect a COI signal. Similarly, we did not observe associations between COI and pregnancy complications or smoking during pregnancy. The distributions of the variables could likewise be an explanation; half of the sample experienced at least one pregnancy complication and most participants did not smoke (87%). Given these distributional concerns, we hesitate to conclude that favorable neighborhood conditions do not influence maternal weight, pregnancy complications, or smoking behavior during pregnancy. Rather, we encourage future work with larger samples and a broader distribution of maternal health experiences to replicate these analyses and consider the role of COI in promoting pregnancy health.

This study has some limitations. The sample size was relatively small, which may have reduced the power to detect associations; the sample size also precluded the possibility of examining patterning of COI associations among subgroups of the population, including racial and ethnic minorities. Moreover, as the complete case analysis excluded more socially vulnerable areas and individuals, the associations reported here likely represent underestimates of the true population associations. In addition, while the Sobel tests provide some preliminary evidence that the observed mediated effect of COI on infant outcomes according to diet was significantly different from zero, Sobel tests do not estimate the magnitude of the mediated effects nor account for the possibility of multiple mediators working simultaneously. We encourage future work to build on this study to identify social and environmental neighborhood assets that could promote healthy pregnancies and favorable infant outcomes in minority communities, and to use mediation methods that can better characterize the pathways linking neighborhood opportunity to maternal and child health.

These limitations notwithstanding, this study has a number of strengths. It is among the first to consider the accumulated impacts of favorable social, environmental, and educational community assets in relation to maternal health during pregnancy and birth size outcomes. We used a multimodal prospective design that included area level measures of neighborhood context, maternal self-reported and physician-assessed clinical characteristics, and directly assessed anthropomorphic measures of infant body size. Moreover, we rigorously controlled for potential confounding by including several sociodemographic and clinical characteristics in the multivariate models.

## 5. Conclusions

This study demonstrated that positive social, educational, and environmental neighborhood attributes cumulatively contributed to healthy pregnancies and favorable birth outcomes. While the identification of individual and community level hazards in relation to perinatal risk is an important public health priority, this work underscores the utility of also considering the accumulated benefit of positive social, environmental, and educational assets for maternal and infant health. Interventions that capitalize on the full range of contextual assets in which mothers live during pregnancy may not only promote healthy births, but may also support child development as well.

## Figures and Tables

**Table 1:** Characteristics of the study population (*n* = 239).

Characteristic	Mean (SD)	% (n)
Child opportunity		
Overall, score	45.69 (32.80)	
Very low		34.31 (82)
Low		14.64 (35)
Moderate		12.13 (29)
High		17.15 (41)
Very high		21.76 (52)
Maternal characteristics		
Age	28.56 (5.57)	
Race, white/not Hispanic		52.72 (126)
Race, not white and/or Hispanic		47.28 (113)
Pre-pregnancy BMI	28.87 (8.67)	
High pregnancy weight gain, yes		51.05 (122)
High pregnancy weight gain, no		48.95 (117)
Depressive symptoms, sum score	8.67 (5.44)	
Pregnancy complications, index	0.67 (0.90)	
Smoked during pregnancy, yes		13.39 (32)
Smoking during pregnancy, no		86.61 (207)
Western diet, sum score	40.06 (15.29)	
Infant characteristics		
Male		49.37 (118)
Female		50.63 (121)
Birth weight, kilograms	3.26 (0.62)	
Birth length, centimeters	48.77 (3.18)	
Birth head circumference, centimeters	33.47 (2.17)	
Birth cephalization index	1.06 (0.22)	
Gestational age at birth	38.89 (1.91)	
Size for gestational age at birth		
Large		7.95 (19)
Small		12.13 (29)
Appropriate		79.92 (191)

**Table 2:** Adjusted associations between neighborhood level child opportunity and birth outcomes.

	Full Sample			Male (*n* = 118)			Female (*n* = 121)		
Outcome	*β*	*SE*	*p*	*β*	*SE*	*p*	*β*	*SE*	*p*
Birth weight, kg	0.003	0.001	0.002	0.003	0.001	0.04	0.003	0.001	0.02
Birth length, cm	0.02	0.005	0.003	0.01	0.01	0.28	0.02	0.01	0.001
Head circumference, cm	0.004	0.004	0.29	0.006	0.006	0.34	0.003	0.005	0.52
Cephalization index	−0.001	0.0003	0.07	−0.0001	0.001	0.90	−0.001	0.0004	0.005
	OR	95% CI		OR	95% CI		OR	95% CI	
Small for gestational age	1.01	0.99	1.03	1.00	0.98	1.02	0.98	0.95	0.99
Large for gestational age	0.99	0.97	1.01	1.01	0.99	1.04	1.01	0.99	1.04

Linear models were adjusted for maternal age, race/ethnicity, smoking during pregnancy, gestational age at delivery, and child sex. Logistic models were adjusted for maternal age, race/ethnicity, smoking during pregnancy, and child sex. Stratified models did not adjust for child sex.

**Table 3:** Adjusted associations between neighborhood level child opportunity and maternal health during pregnancy.

Maternal Health	β	SE	*p*
Pregnancy complications index	−0.004	0.002	0.82
Pre-pregnancy BMI	−0.03	0.02	0.15
Depressive symptoms	−0.03	0.01	0.02
Western diet	−0.09	0.03	0.0003
	OR	95% CI	
Smoked	0.99	0.98	1.01
High weight gain in pregnancy	1.00	0.99	1.004

All models adjusted for maternal age and race/ethnicity. Each maternal health outcome was treated independently; parameter estimates were not adjusted for other factors listed in the table.

**Table 4:** Associations between domains of neighborhood level child opportunity and maternal and infant outcomes.

	Educational			Health and Environmental			Social and Economic		
Outcome	β	SE	*p*	β	SE	*p*	β	SE	*p*
Birth weight	0.004	0.001	<0.001	0.003	0.001	0.01	0.002	0.001	0.02
Birth length	0.02	0.006	0.004	0.02	0.006	0.01	0.01	0.01	0.001
Cephalization index	−0.001	0.0003	0.03	−0.001	0.0003	0.02	−0.0004	0.0003	0.23
Maternal depression	−0.02	0.015	0.28	−0.03	0.013	0.03	−0.03	0.01	0.01
Maternal western diet	−0.11	0.03	0.0005	−0.05	0.02	0.03	−0.09	0.03	0.0005

Infant models were adjusted for maternal age, race/ethnicity, smoking during pregnancy, child sex, and gestational age at delivery. Maternal models were adjusted for age and race/ethnicity.

## Data Availability

The data presented in this study are available on request from the corresponding author. The data are not publicly available in order to maintain participant confidentiality.

## Supplementary Materials

The following are available online at https://www.mdpi.com/article/10.3390/ijerph18116161/s1, Table S1: Child Opportunity Index (COI) domains and indicators, Table S2: adjusted associations between COI and birth outcomes not controlling for gestational age, and Table S3: inputs and Sobel tests to determine whether maternal depression or diet during pregnancy mediate the association between COI and birth outcomes.
