# Supplementary material for: Prenatal Exposure to Favorable Social and Environmental Neighborhood Conditions Is Associated with Healthy Pregnancy and Infant Outcomes
Source: Int J Environ Res Public Health. 2021 Jun 7;18(11):6161. doi: 10.3390/ijerph18116161 (PMC8200992; doi:10.3390/ijerph18116161)
Supplement: Supplementary file 1 [file ijerph-18-06161-s001.zip › ijerph-1230181-supplementary.pdf]

Supplemental Table S1. Child Opportunity Index (COI) domains and indicators

Domain	Indicator	Description (source)
Education	Early childhood education (ECE) centers	Number of ECE centers within a 5-mile radius (data collection from state and federal sources)
	High-quality ECE centers	Number of NAEYC accredited centers within a 5-mile radius (data collection from state and federal sources)
	ECE enrollment	Percent 3- and 4-year-olds enrolled in nursery school, preschool or kindergarten (ACS)
	Third grade reading proficiency	Percent third graders scoring proficient on standardized reading tests, converted to NAEP scale score points (EDFacts, GS and SEDA)
	Third grade math proficiency	Percent third graders scoring proficient on standardized math tests, converted to NAEP scale score points (EDFacts, GS and SEDA)
	High school graduation rate	Percent ninth graders graduating from high school on time (EDFacts and GS)
	Advanced Placement (AP) course enrollment	Ratio of students enrolled in at least one AP course to the number of 11th and 12th graders (CRDC)
	College enrollment in nearby institutions	Percent 18-24 year-olds enrolled in college within 25-mile radius (ACS)
	School poverty	Percent students in elementary schools eligible for free or reduced-price lunches, reversed (NCES CCD)
	Teacher experience	Percent teachers in their first and second year, reversed (CRDC)
	Adult educational attainment	Percent adults ages 25 and over with a college degree or higher (ACS)
Health & Environment	Access to healthy food	Percent households without a car located further than a half-mile from the nearest supermarket, reversed (USDA)
	Access to green space	Percent impenetrable surface areas such as rooftops, roads, or parking lots, reversed (CDC)
	Walkability	EPA Walkability Index (EPA)
	Housing vacancy rate	Percent housing units that are vacant, reversed (ACS)
	Hazardous waste dump sites	Average number of Superfund sites within a 2-mile radius, reversed (EPA)
	Industrial pollutants in air, water or soil	Index of toxic chemical released by industrial facilities, reversed (EPA)

	Airborne microparticles	Mean estimated microparticle (PM2.5) concentration, reversed (CDC)
	Ozone concentration	Mean estimated 8-hour average ozone concentration, reversed (EPA)
	Extreme heat exposure	Summer days with maximum temperature above 90F, reversed (CDC)
	Health insurance coverage	Percent individuals ages 0-64 with health insurance coverage (ACS)
Social & Economic	Employment rate	Percent adults ages 25-54 who are employed (ACS)
	Commute duration	Percent workers commuting more than one hour one way, reversed (ACS)
	Poverty rate	Percent individuals living in households with incomes below 100% of the federal poverty threshold, reversed (ACS)
	Homeownership rate	Percent owner-occupied housing units (ACS)
	High-skill employment	Percent individuals ages 16 and over employed in management, business, financial, computer, engineering, science, education, legal, community service, health care practitioner, health technology, arts and media occupations (ACS)
	Median household income	Median income of all households (ACS)
	Single-headed households	Percent family households that are single-parent headed, reversed (ACS)

Abstracted from: Noelke, C., McArdle, N., Baek, M., Huntington, N., Huber, R., Hardy, E., & Acevedo-Garcia, D. (2020). Child Opportunity Index 2.0 Technical Documentation. Retrieved from diversitydatakids.org/research- library/research-brief/how-we-built-it.

Abbreviations: ACS = American Community Survey; CDC = Centers for Disease Control and Prevention; CRDC = Civil Rights Data Collection; EPA = Environmental Protection Agency; EDFacts = U.S. Department of Education EDFacts Data ; GS = GreatSchools; NCES CCD = National Center for Health Statistics Common Core of Data; SEDA = Stanford Education Data Archive; USDA = United States Department of Agriculture

Supplemental Table S2. Adjusted associations between neighborhood level child opportunity and birth outcomes, with and without controlling for gestational age (GA)

	Model set 1: Controlled for GA			Model set 2: Not controlled for GA		
Outcome	*β*	*SE*	*p*	*β*	*SE*	*p*
Gestational age	-	-	-	-0.005	0.004	0.19
Birth weight	0.003	0.001	0.002	0.002	0.001	0.05
Birth length	0.02	0.005	0.003	0.01	0.006	0.05
Head circumference	0.004	0.004	0.29	0.001	0.004	0.85
Cephalization index	-0.001	0.0003	0.07	-0.001	0.0005	0.04

All models were adjusted for maternal age, race/ethnicity, smoking during pregnancy, and child sex. Model set 1 additionally controls for gestational age at delivery.

Supplemental Table S3. Inputs and Sobel tests to determine whether maternal depression or diet during pregnancy mediate the association between COI and birth outcomes

	COI → Mediator [a path] β (SE)	Mediator → Outcome; COI in the model [b path] β (SE)	Sobel test (SE)	p-value
Birth weight				
Depression	-0.03 (0.01)	-2.19 (7.85)	0.28 (0.27)	0.78
Diet	-0.11 (0.0)	-7.18 (2.67)	2.33 (0.33)	0.02
Birth length				
Depression	-0.03 (0.01)	-0.07 (0.04)	1.37 (0.002)	0.17
Diet	-0.11 (0.02)	-0.04 (0.02)	1.99 (0.005)	0.045
Cephalization				
Depression	-0.03 (0.01)	-0.001 (0.003)	0.43 (0.0001)	0.66
Diet	-0.11 (0.02)	0.002 (0.001)	-1.46 (0.0001)	0.14

Notes. The Sobel test statistic is calculated with this formula: z-value = $a*b$/SQRT($b^2*s_a^2 + a^2*s_b^2$), where a= regression coefficient for the association between the independent variable and mediator (e.g., COI predicting maternal factor), and b= regression coefficient for the association between the mediator and the dependent variable, with the independent variable in the model (Maternal factor predicting infant outcome, COI in the model as a covariate).
